# Efficacy of pegylated interferon alpha in treating postpartum women with chronic hepatitis B virus infection: a multicenter prospective study

**DOI:** 10.3389/fcimb.2026.1802657

**Published:** 2026-04-29

**Authors:** Hongmei Gong, Ming Liu, Xuqing Zhang, Wei Sun, Hongli Deng, Wei Liu, Yi Wu, Guohong Deng, Jie Xia, Qing Mao, Li Jiang

**Affiliations:** 1 Department of Infectious Diseases, The First Affiliated Hospital of Army Medical University, Chongqing, China; 2 Department of Infectious Diseases, The Third Affiliated Hospital of Chongqing Medical University (Fangda Hospital), Chongqing, China; 3 Department of Infectious Diseases and Hepatology, People’s Hospital of Banan District, Chongqing, China; 4 Department of Infectious Diseases, People’s Hospital of Hechuan District, Chongqing, China; 5 Department of Infectious Diseases, Jiangbei Branch of The First Affiliated Hospital of Army Medical University, Chongqing, China

**Keywords:** antiviral therapy, functional cure, HBsAg clearance, HBV-infected postpartum women, pegylated interferon

## Abstract

**Objective:**

Hepatitis B surface antigen (HBsAg) clearance is a key milestone for functional (clinical) cure of chronic hepatitis B virus (HBV) infection. However, data on the efficacy and safety of pegylated interferon alpha (Peg-IFNα) for HBsAg clearance in postpartum women with chronic HBV infection remain scarce. This study aimed to prospectively evaluate the efficacy and safety of Peg-IFNα in promoting HBsAg clearance in this specific population.

**Methods:**

A total of 263 HBV-infected postpartum women with a baseline HBsAg levels ≤3000 IU/mL were enrolled. Based on patient preference, they were assigned to either the Peg-IFNα group (n=62, receiving Peg-IFNα monotherapy or combination therapy with nucleos(t)ide analogs [NAs]) or the control group (n=201, receiving NAs or follow-up only). The duration of Peg-IFNα treatment was ≥48 weeks. Propensity score matching (PSM) was performed to balance baseline characteristics. The primary endpoints were both HBsAg clearance rate and the magnitude of HBsAg decline.

**Results:**

At week 48, the Peg-IFNα group had significantly higher HBsAg clearance (29.03% [18/62]) and seroconversion rates (27.42% [17/62]) than the control group (both 0%, P < 0.001). Post-PSM, the Peg-IFNα group still had a significantly higher HBsAg clearance/seroconversion rate (30.43% [14/46]) versus the control group (0%, P < 0.001). Subgroup analysis showed that patients with baseline HBsAg < 1000 IU/mL had higher clearance rates (pre-PSM: 39.02% [16/41]; post-PSM: 40.00% [12/30]). Receiver operating characteristic (ROC) curve analysis identified a ≥ 99.2% HBsAg reduction from baseline to week 12 as a strong predictor of week 48 HBsAg clearance (AUC ≥0.9; sensitivity =0.818; specificity= 1.000).

**Conclusion:**

Peg-IFNα treatment can achieve high rates of HBsAg clearance and seroconversion in postpartum women with chronic HBV infection. The postpartum period represents a unique and favorable immune window for interferon-based intervention, and patients with baseline HBsAg <1000 IU/mL derive particular benefit from this strategy.

## Highlights

- Functional (clinical) cure is the primary therapeutic goal for chronic HBV infection, with HBsAg clearance as its key endpoint.
- The postpartum period represents a unique immune activation window for pursuing clinical cure in women with chronic HBV infection.
- Postpartum antiviral therapy with pegylated interferon achieves favorable HBsAg clearance rates, particularly in patients with low baseline HBsAg.
- Early on-treatment HBsAg reduction at week 12 is a robust predictor of subsequent HBsAg clearance in this population.

## 1 Introduction

Hepatitis B virus (HBV) infection remains a major global public health issue. The World Health Organization (WHO) estimates a global hepatitis B surface antigen (HBsAg) seroprevalence of 3.2%, with approximately 254 million individuals chronically infected and roughly 820, 000 annual deaths attributed to HBV-related diseases (Yan et al., 2025). In China, it is estimated that there are still 75 million people living with HBV (Hui et al., 2024), with mother-to-child transmission (MTCT) as the primary infection route and a key driver of chronicity (Chinese Society of Hepatology, Chinese Medical Association; Chinese Society of Infectious Diseases, Chinese Medical Association, 2022). The HBsAg positivity rate among Chinese pregnant women is 6.3% (Cui et al., 2018), posing significant challenges to maternal-child health and public health (Huang et al., 2024). Antiviral therapy for pregnant women with high viral loads effectively reduces MTCT risk (Infectious Diseases Physicians Branch, Chinese Medical Doctor Association; Chinese Society of Infectious Diseases, Chinese Medical Association, 2024). However, 25%–45% of women with chronic HBV infection develop hepatitis flares or viral reactivation postpartum (Song et al., 2022), which may progress to severe hepatitis (Song, 2022)-highlighting the critical need for sustained postpartum management.

Antiviral therapy is the cornerstone of management for patients with chronic hepatitis B (CHB), aiming to delay disease progression and reduce of adverse outcomes, thereby improving patients’ quality of life. Since the complete elimination of covalently closed circular DNA (cccDNA) and integrated HBV DNA remains a formidable challenge, functional (clinical) cure has emerged as the most pragmatic therapeutic goal at the current stage (Vittal et al., 2022; Song et al., 2021a). This is defined as sustained HBsAg loss (≤ 0.05 IU/mL) and HBV DNA less than the lower limit of quantitation (LLOQ)24 weeks off-treatment with or without seroconversion to anti-HBs, accompanied by sustained HBeAg loss and normalization of alanine aminotransferase (ALT) levels (Vittal et al., 2022; Song et al., 2021a; Lok, 2024). Achieving this milestone is associated with a significant reduction in the incidence of decompensated cirrhosis, hepatocellular carcinoma (HCC), and liver-related mortality (Vittal et al., 2022; Kaur et al., 2022).

HBsAg clearance is a key determinant to functional cure and should be prioritized in eligible patients. Pegylated interferon alpha (Peg-IFNα) demonstrates superior efficacy in specific populations, exhibiting HBsAg clearance rates of 33.8% in nucleos(t)ide analog (NA)-experienced patients with baseline HBsAg <1500 IU/mL (Author Group of Expert Consensus, 2025), 47% in inactive carriers (Song et al., 2021b), and 38.98% in children aged 1–16 years (Zhang M. et al., 2024). However, HBV-infected postpartum women represent a unique patient cohort. While current guidelines primarily focus on preventing mother-to-child transmission (MTCT), optimal postpartum antiviral strategies remain controversial (Ghany et al., 2025). Previous studies suggest that continuing NA therapy postpartum can reduce HBsAg and HBeAg levels (Pan et al., 2012; Feng et al., 2023). Furthermore, in women who received NAs during the third trimester, the addition of Peg-IFNα postpartum therapy achieved an HBsAg clearance rate of 26.7% (Lu et al., 2015). Notably, among HBeAg-negative postpartum women with baseline HBsAg levels ≤3000 IU/mL, 48 weeks of Peg-IFNα therapy yielded HBsAg clearance and seroconversion rates of 51.06% and 40.43%, respectively (Zhong et al., 2024). These findings highlight the postpartum period as a potential “window of immune activation” conducive to achieving functional cure (Han and Jiang, 2024).

Nevertheless, prospective data on HBV-infected postpartum women remain limited due to their unique physiological status. This study is the first to assess the efficacy and safety of Peg-IFNα therapy for HBsAg clearance in this population using a multicenter prospective cohort study design, offering real-world evidence to inform optimal postpartum management.

## 2 Materials and methods

### 2.1 Study population

This prospective real-world study, registered as the Sunshine Program (ChiCTR2200058096; Ethics Approval No: [B] KY2021095), enrolled postpartum women aged 20–45 years with CHB at 6–48 weeks postpartum (median enrollment time 45 [33, 47] weeks). Participants were recruited from infectious disease outpatient clinics across participating centers between April 2022 and March 2024. All provided written informed consent. The inclusion criteria were as follows: baseline HBsAg levels ≤3000 IU/mL; ALT level <10 × upper limit of normal (ULN); no restrictions on HBeAg or HBV DNA status; non-lactating status; and no pregnancy plans within 2 years. The exclusion criteria included liver cirrhosis or other concomitant liver diseases; severe systemic comorbidities, coinfection with other hepatotropic viruses, hypersensitivity to Peg-IFNα, and being deemed ineligible for Peg-IFNα therapy by the investigator.

### 2.2 Research methods

Patients were grouped per their treatment preference: the Peg-IFNα group included two subgroups-patients who received NAs during pregnancy with the addition of Peg-IFN α-2b (Pegbina; Xiamen Amoytop Biotech, 180 μg/week, subcutaneous injection), and patients without NA exposure during pregnancy who received Peg-IFN α-2b monotherapy or combination therapy with tenofovir disoproxil fumarate (TDF, 300 mg/day, oral administration), for HBV DNA levels of 2–5 log_10_ IU/mL).The control group included patients who continued their original NA regimen or only underwent regular observation and follow-up. The total duration of Peg-IFNα-based therapy was at least 48 weeks; consolidation therapy was administered at the physician’s discretion after HBsAg clearance; and treatment was discontinued if HBsAg levels showed no significant reduction from baseline. The primary endpoints were both HBsAg clearance rate and the magnitude of HBsAg decline at week 48 of treatment. Secondary endpoints included the week-48 HBsAg clearance rate in the subgroup with baseline HBsAg levels < 1000 IU/mL and the predictors of week-48 HBsAg clearance. Adverse events associated with Peg-IFNα were closely monitored throughout the study to evaluate treatment safety.

### 2.3 Research evaluation

All enrolled patients underwent comprehensive assessments, including hepatitis B virus marker (HBVM), HBV DNA quantification, liver function tests (LFTs), renal function tests, blood glucose measurement, complete blood count (CBC), thyroid function tests, and liver disease-related autoantibody tests. Upper abdominal ultrasound and FibroScan examinations were also performed.

During treatment, CBC, LFTs, renal function, and blood glucose levels were assessed every 4 weeks. HBVM, COBAS HBV DNA quantification, thyroid function, and liver disease-related autoantibodies were assessed every 12 weeks. HBVM was measured using an ARCHITECT i2000sr automated immunoassay analyzer (Chemiluminescent Microparticle Immunoassay, Abbott Diagnostics, Ireland). The detection range of HBsAg levels was 0.05–250, 000 IU/mL (after dilution). HBsAg clearance was defined as ≤0.05 IU/mL, and anti-HBs positivity was defined as > 10 IU/mL. Serum HBV DNA was quantified using the Roche Cobas TaqMan fully automated PCR system (Roche Diagnostics, Switzerland), with a lower LLOQ of 20 IU/mL. Serum ALT levels were measured using a HITACHI 7600–020 fully automated biochemical analyzer (Hitachi High-Technologies Corporation, Japan), with a normal range of 0–42 IU/L. Safety assessments included monitoring for adverse events such as fever, myalgia, fatigue, alopecia, psychiatric disorders, anorexia, neutropenia, elevated ALT levels, thrombocytopenia, and thyroid dysfunction.

### 2.4 Propensity score matching method

Propensity score matching (PSM) was performed using SPSS 26.0 software. Receiving Peg-IFNα therapy was set as the dependent variable, and age, use of NAs, baseline ALT level, baseline HBsAg level, baseline HBV DNA level, and baseline HBeAg status were included in the propensity score model. A 1:1 nearest-neighbor matching algorithm was applied with a caliper width of 0.02 and without replacement. The balance of covariates was evaluated using standardized mean differences (SMD), with SMD < 0.2 indicating satisfactory balance between groups.

### 2.5 Statistical analysis

Data were analyzed using SPSS 26.0 software. Categorical variables were presented as frequencies (%). Continuous variables with normal distribution were expressed as mean ± SD, while non-normally distributed variables were expressed as median (interquartile range, P_25_–P_75_). The χ² test was used for between-group comparisons.

Since patients were only evaluated at fixed time points (12, 24, and 48 weeks) and the exact time of HBsAg clearance could not be determined, Kaplan–Meier analysis was not performed. Instead, HBsAg clearance rates at week 24 and week 48 were used to compare therapeutic efficacy. Logistic regression and ROC curve analysis were applied to identify predictive factors for HBsAg clearance. A two-sided P < 0.05 was considered statistically significant.

## 3 Results

### 3.1 Baseline characteristics

**Figure 1:**
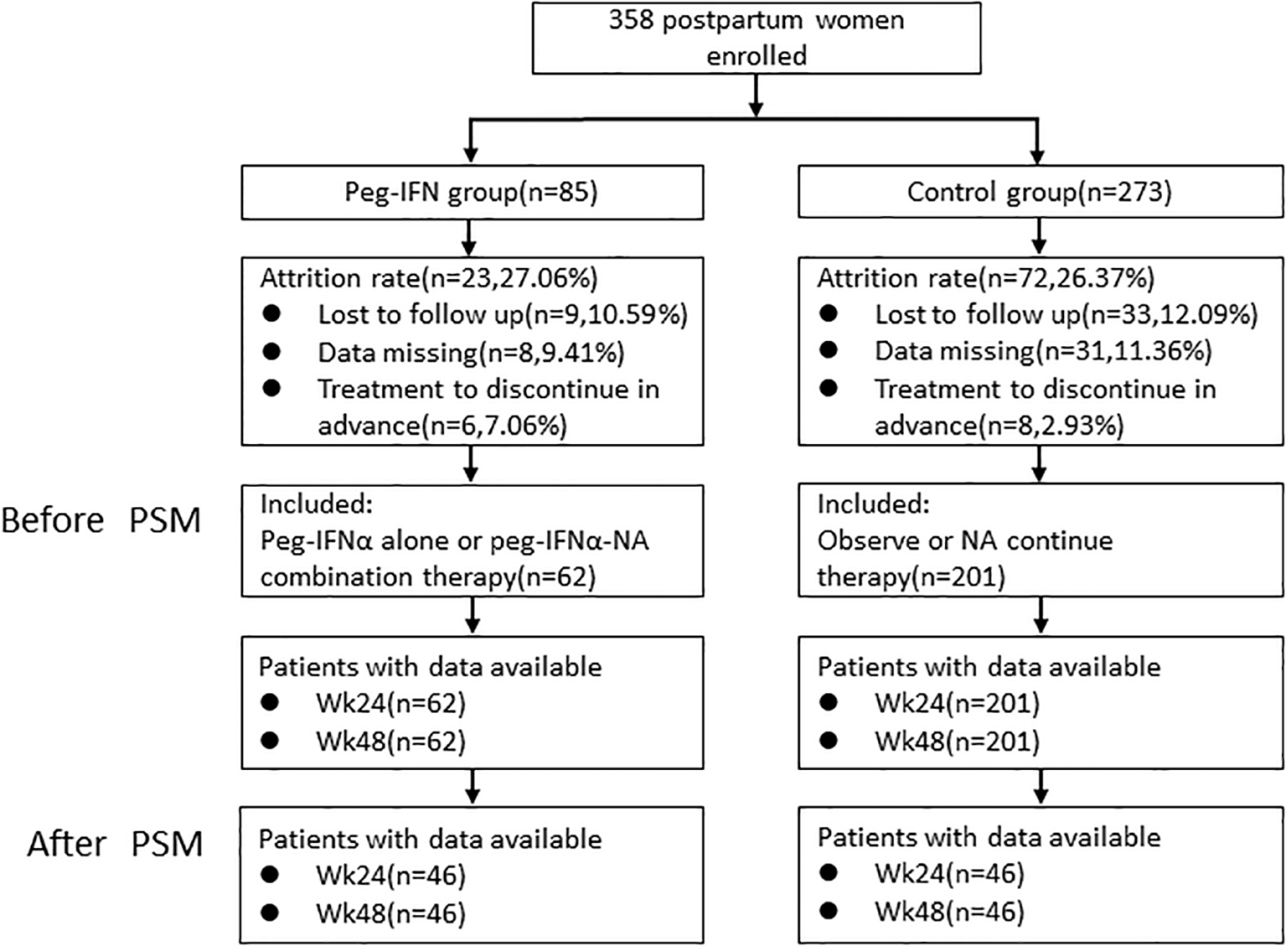
Flow diagram of patient enrollment.

A total of 358 postpartum women with chronic HBV infection were enrolled, including 85 in the Peg-IFNα group and 273 in the control group. During follow-up, 23 patients (27.06%) were excluded from the Peg-IFNα group: 9 (10.59%) lost to follow-up, 8 (9.41%) with missing data, and 6 (7.06%) discontinuing treatment early (4 for adverse events, 2 for poor compliance). In the control group, 72 patients (26.37%) were excluded: 33 (12.09%) lost to follow-up, 31 (11.36%) with missing data, and 8 (2.93%) discontinuing early (3 for adverse events, 5 for poor compliance). The overall dropout rate and distribution of reason did not differ significantly between groups (all P>0.05), suggesting no substantial selection bias due to attrition. Finally, 62 patients in the Peg-IFNα group and 201 in the control group were included in the pre-PSM analysis, and 46 patients per group included after 1:1 propensity score matching (**Figure 1**). There were significant differences in age, ALT and HBeAg status at baseline before PSM (p < 0.05). All baseline variables were well balanced with no significant differences after PSM (p> 0.05) (**Table 1**).

**Table 1:** Baseline characteristics of the study population.

Variables		Before PSM							After PSM		
	Peg-IFNα group N=62		Control group N=201	SMD	P	Peg-IFNα group N=46		Control group N=46		SMD	P
Age, mean ± SD	32.13 ± 3.55		29.19 ± 4.04	0.656	0.000	31.78 ± 3.27		31.91 ± 3.73		0.037	0.859
Use of NAs, n (%)	44 (70.97)		145 (72.14)	0.018	0.858	35 (76.09)		38 (82.61)		0.162	0.440
ALT, IU/L, M (P_25_, P_75_)	20.75 (16.28, 24.98)		29.70 (21.33, 44.15)	0.486	0.000	20.80 (16.43, 25.80)		15.90 (13.15, 27.35)		0.175	0.453
HBsAg (log_10_ IU/mL), mean ± SD	2.68 ± 0.68		2.82 ± 0.68	0.181	0.161	2.66 ± 0.71		2.78 ± 0.56		0.188	0.369
HBV DNA detectable[2~5 log_10_ IU/mL], n (%)	18 (29.03)		42 (20.90)	0.189	0.182	11 (23.91)		12 (26.09)		0.050	0.810
HBeAg positive, n (%)	9 (14.52)		86 (42.79)	0.658	0.000	8 (17.39)		11 (23.91)		0.162	0.440

SD, standard deviation; PSM, propensity score matching; SMD, Standardized mean differences; ALT, alanine aminotransferase; HBeAg, hepatitis B e antigen; HBsAg, hepatitis B surface antigen; NAs, nucleos (t)ide analogs; Peg-IFN, pegylated interferon α.

### 3.2 Peg-IFNα-based therapy significantly increases HBsAg clearance rates compared with control treatment

**Figure 2:**
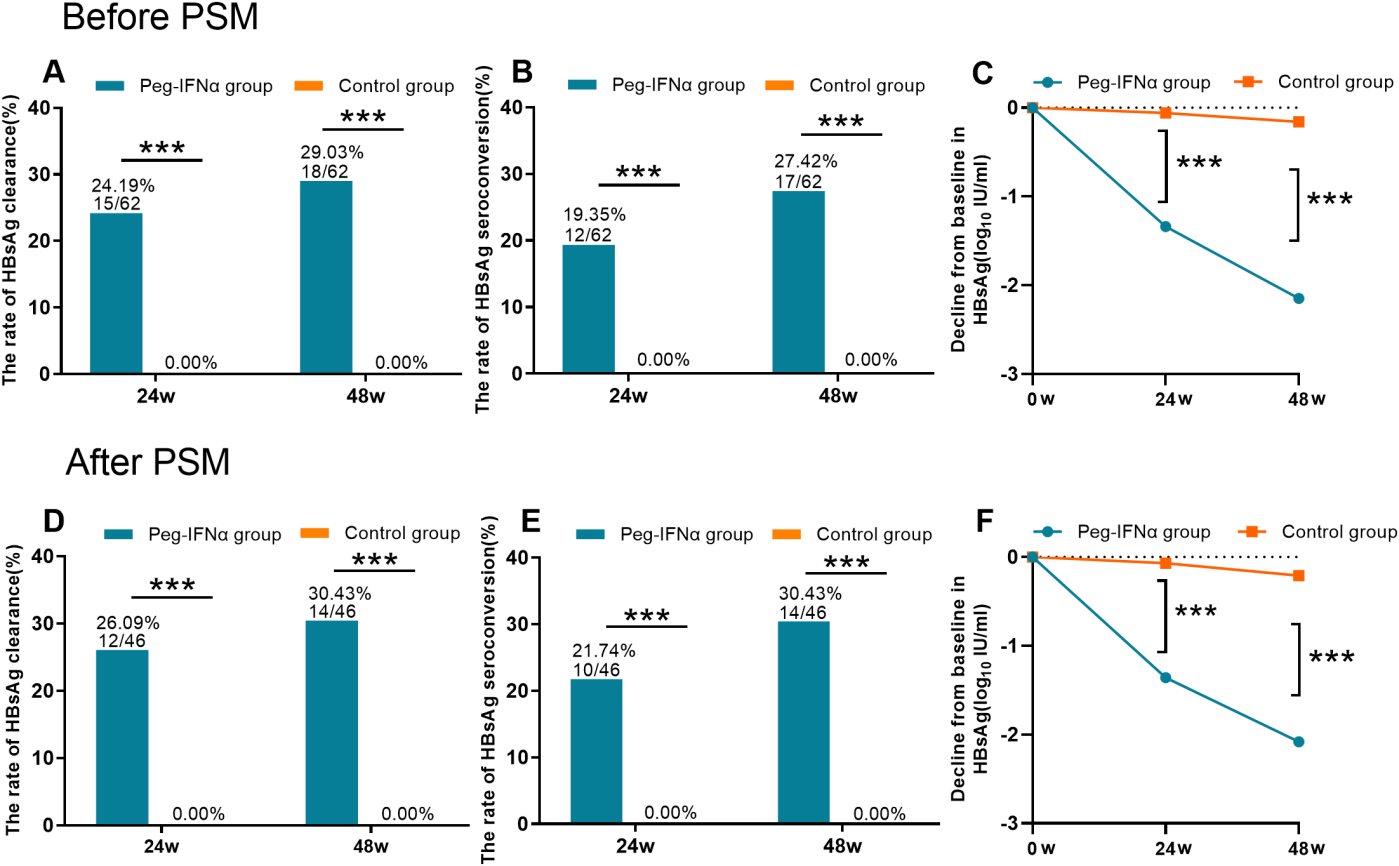
HBsAg clearance and seroconversion rates in postpartum women with chronic HBV infection receiving Peg-IFNα therapy. Peg-IFNα treatment yielded significantly higher HBsAg clearance and seroconversion rates at week 48 (29.03% and 27.42%, respectively) compared with controls [0% and 0%; both P<0.001; **(A, B)**]. HBsAg decline was also markedly greater in the Peg-IFNα group at weeks 24 and 48 **(C)**. After PSM, significant between-group differences were sustained for HBsAg clearance and seroconversion (30.43% vs. 0% for both; **(D, E)**] and HBsAg reduction [**(F)**; all P<0.001]. *** p < 0.001.

At week 48, the Peg-IFNα group exhibited significantly higher HBsAg clearance (29.03%, 18/62) and seroconversion rates (27.42%, 17/62) compared with the control group (both 0%, P < 0.001; **Figures 2A, B**). The magnitude of HBsAg decline was also markedly greater in the Peg-IFNα group at weeks 24 and 48: 1.13 (0.27, 2.38) vs. 0.02 (0.01, 0.08) and 2.02 (1.09, 3.22) vs. 0.09 (0.06, 0.18) log_10_ IU/mL, respectively (both P < 0.001; **Figure 2C**). After PSM, the between-group differences remained significant, with HBsAg clearance and seroconversion rates of 30.43% (14/46) in the Peg-IFNα group versus 0% in controls (both P < 0.001; **Figures 2D, E**). HBsAg decline was also consistently larger in the Peg-IFNα group at weeks 24 and 48: 1.14 (0.25, 2.39) vs. 0.03 (0.00, 0.10) and 1.99 (0.71, 3.52) vs. 0.15 (0.12, 0.22) log_10_ IU/mL, respectively (both P < 0.001; **Figure 2F**).

The HBeAg seroconversion rate was significantly higher in the Peg-IFNα group than in the control group, both pre-PSM (55.56%, 5/9 vs. 0%, 0/86; P < 0.001) and post-PSM (50.00%, 4/8 vs. 0%, 0/15; P < 0.05). Additionally, all HBV DNA-positive patients in the Peg-IFNα group achieved a complete virological response (CVR), and the CVR rate at week 48 was significantly higher in the Peg-IFNα group than in the control group: pre-PSM (100%, 62/62 vs. 80.60%, 162/201; P < 0.001) and post-PSM (100%, 46/46 vs. 84.78%, 39/46; P < 0.05). There was no statistically significant difference in the HBsAg clearance rate between the Peg-IFNα plus NA combination subgroup and the Peg-IFNα monotherapy subgroup within the Peg-IFNα cohort, either pre-PSM (29.55%, 13/44 vs. 27.78%, 5/18; P = 0.889) or post-PSM (31.43%, 11/35 vs. 27.27%, 3/11; P = 0.794).

### 3.3 Patients with baseline HBsAg < 1000 IU/mL achieve higher HBsAg clearance rates

**Figure 3:**
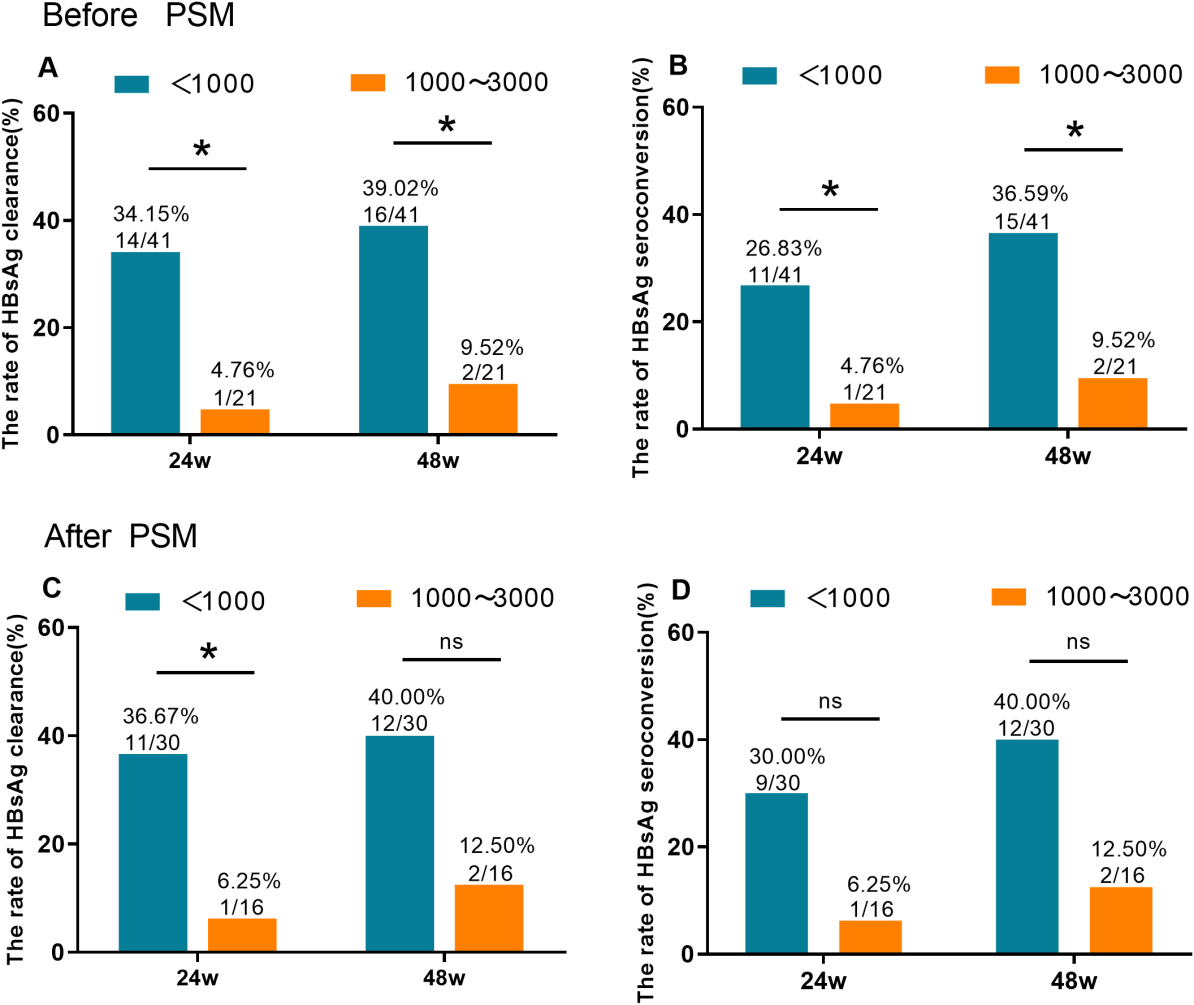
HBsAg clearance and seroconversion based on baseline HBsAg stratification in postpartum women with chronic HBV infection receiving Peg-IFNα therapy. At week 48 of treatment **(A, B)**, before PSM, the HBsAg clearance rate (39.02%) and seroconversion rate (36.59%) in the group with baseline HBsAg < 1000 IU/mL were significantly higher than those in the group with 1000 ≤ HBsAg < 3000 IU/mL (9.52% and 9.52%, respectively; P < 0.05). After PSM **(C, D)**, the HBsAg clearance rate (40.00%) and seroconversion rate (40.00%) in the group with baseline HBsAg < 1000 IU/mL were higher than those in the group with 1000 ≤ HBsAg < 3000 IU/mL (12.50% and 12.50%, respectively), but the differences were not statistically significant (ns, P > 0.05). * p < 0.05.

A subgroup analysis was conducted in the Peg-IFNα cohort, stratified by baseline HBsAg levels (<1000 IUmL vs. 1000 ≤ HBsAg < 3000 IU/mL). At week 48, patients with baseline HBsAg < 1000 IU/mL had significantly higher HBsAg clearance rates (39.02%, 16/41) and seroconversion rates (36.59%, 15/41) than those with 1000 ≤ HBsAg <3000 IU/mL (9.52%, 2/21 for both; P < 0.05; **Figures 3A, B**). After PSM, patients with baseline HBsAg < 1000 IU/mL had numerically higher clearance rates (40.00%, 12/30) and seroconversion rates (40.00%, 12/30) than those with 1000 ≤ HBsAg < 3000 IU/mL (12.50%, 2/16 for both), but the difference did not reach statistical significance (P = 0.054; **Figures 3C, D**).

### 3.4 HBsAg clearance rate from baseline to week 12 predicts HBsAg clearance and seroconversion at week 48

**Figure 4:**
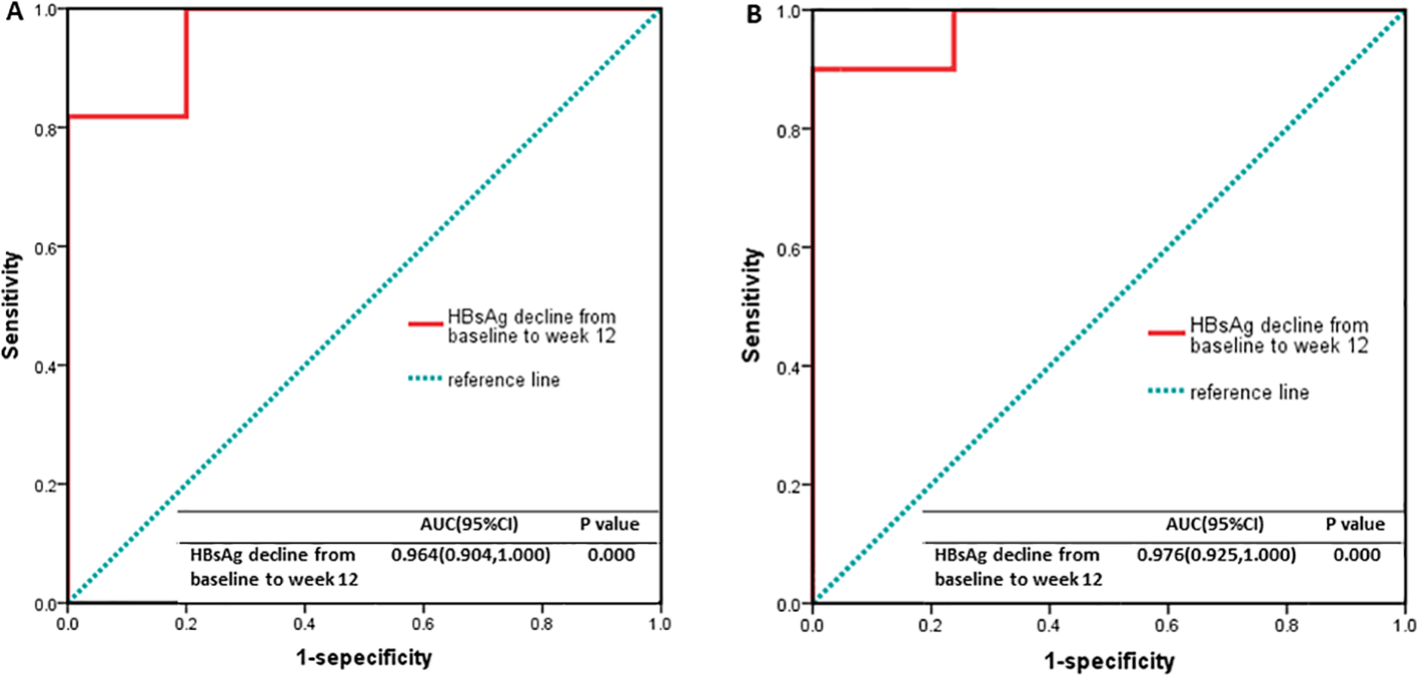
Receiver operating characteristic curves. **(A)** For HBsAg clearance: The area under the curve (AUC) was ≥0.9. The optimal cutoff value was 99.12%, with a sensitivity of 0.818 and a specificity of 1.000. **(B)** For HBsAg seroconversion: The AUC ≥0.9. The optimal cutoff value was 99.12%, with a sensitivity of 0.900 and a specificity of 1.000.

Factors associated with HBsAg clearance at week 48 in the Peg-IFNα group were analyzed. Univariate logistic regression analysis revealed that baseline HBsAg level, week 12 HBsAg level, and week 12 HBsAg reduction rate were significant predictors (P < 0.05). Multivariate logistic regression analysis confirmed that a reduction in HBsAg levels at week 12 was an independent predictor (P < 0.05; **Table 2**). ROC curve analysis demonstrated that a week 12 HBsAg reduction rate ≥ 99.2% strongly predicted HBsAg clearance at week 48 (area under the curve [AUC] ≥ 0.9; sensitivity, 0.818; specificity, 1.000; **Figure 4A**) and seroconversion at week 48 (AUC ≥ 0.9; sensitivity, 0.900; specificity, 1.000; **Figure 4B**).

**Table 2:** Factors associated with HBsAg clearance at week 48 of Peg-IFNα therapy.

Predictors	Univariate analysis		Multivariate analysis	
	OR (95%CI)	P	OR (95%CI)	P
Age, years	0.932 (0.765~1.136)	0.486		
Use of NAs	0.740 (0.505~1.085)	0.123		
Baseline HBsAg level, IU/ml	0.999 (0.997~1.000)	0.034 *	1.000 (0.997~1.002)	0.736
Baseline HBsAg level <1000 IU/ml	3.474 (0.653~18.471)	0.144		
Baseline HBV DNA, <20 IU/ml	4.174 (0.466~37.403)	0.202		
Baseline ALT level, U/L	1.046 (0.962~1.137)	0.296		
ALT level at week 12, U/L	1.001 (0.993~1.008)	0.884		
ALT level at week 24, U/L	0.989 (0.972~1.007)	0.248		
HBsAg at week 12, IU/ml	0.994 (0.989~0.999)	0.029 *	1.003 (0.998~1.008)	0.175
HBsAg at week 24, IU/ml	0.788 (0.579~1.073)	0.131		
HBsAg decline from baseline to week 12, %	1.115 (1.036~1.200)	0.004 *	1.166 (1.022~1.331)	0.022 *
HBsAg decline from baseline to week 24, %	0.169 (0.654~1.213)	0.117		

Univariate logistic regression analysis revealed that baseline HBsAg level, week 12 HBsAg level, and week 12 HBsAg reduction rate were significant predictors of HBsAg clearance at week 48 with Peg-IFN α-2b therapy (P < 0.05). Multivariate logistic regression analysis revealed that. week-12 HBsAg reduction was an independent predictor of HBsAg clearance at week 48 with Peg-IFN α-2b therapy (P < 0.05). *P < 0.05.

### 3.5 Follow-up

Peg-IFNα group: At 24 weeks off-treatment (n=50), one additional patient achieved HBsAg clearance and one achieved seroconversion, with no HBsAg reversion observed. The cumulative HBsAg clearance rate was 32.00% (16/50). At 48 weeks off -treatment (n=37), no new cases of HBsAg clearance or seroconversion were recorded, and one case of HBsAg reversion was noted. Control group: At 48 weeks off -treatment (n=201), no new cases of HBsAg clearance or seroconversion were recorded.

Peg-IFNα group (post-PSM): At 24 weeks off -treatment (n=36), one additional patient achieved HBsAg clearance and one achieved seroconversion, with no HBsAg reversion observed. The cumulative HBsAg clearance rate was 33.33% (12/36). At 48 weeks off-treatment (n=27), no new cases of HBsAg clearance or seroconversion were recorded, and no HBsAg reversion was observed.

**Figure 5:**
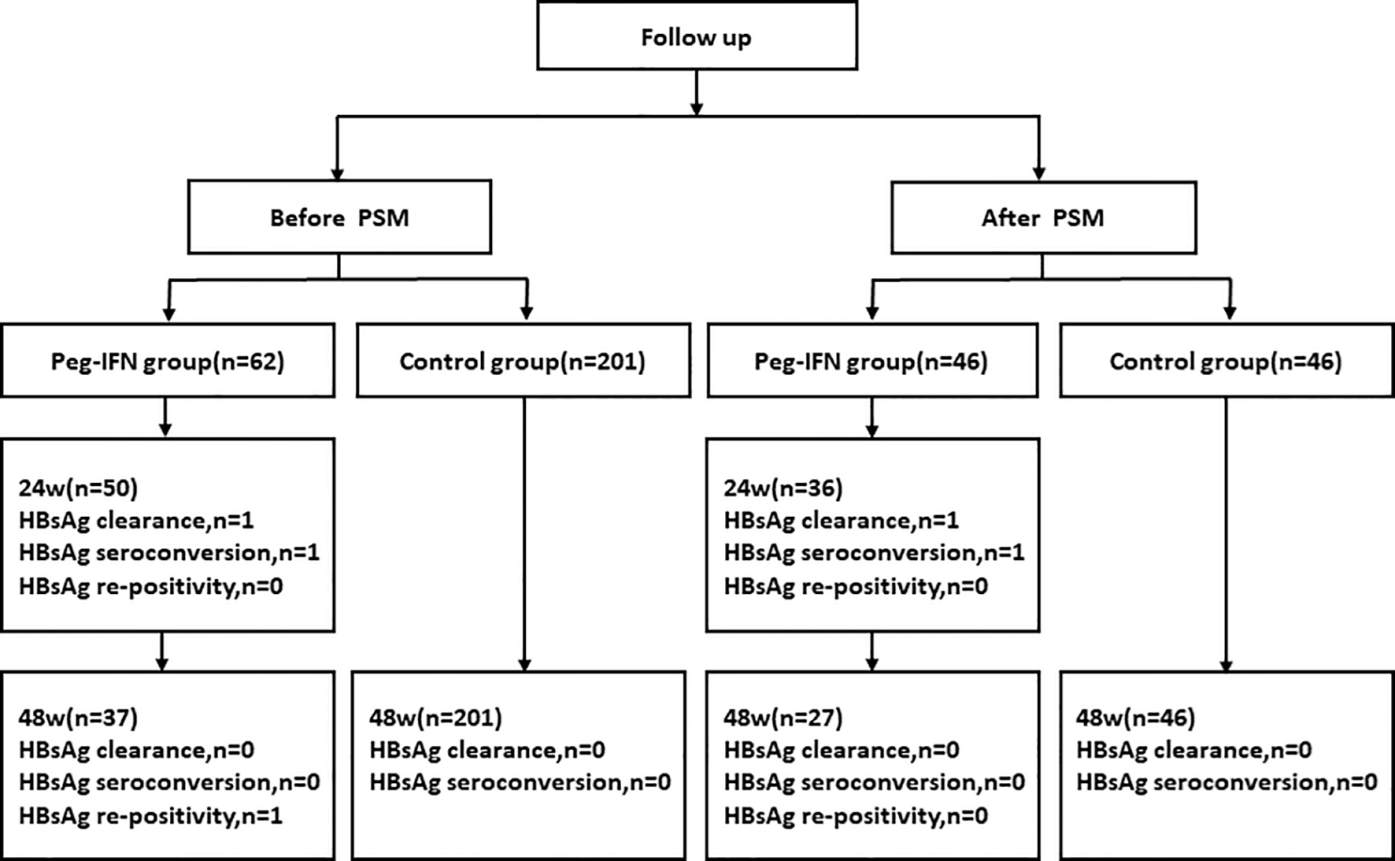
Patient follow-up diagram.

Control group (post-PSM): At 48 weeks off-treatment (n=46), no new cases of HBsAg clearance or seroconversion were recorded (**Figure 5**).

### 3.6 Adverse events

Common adverse events (AEs) observed in the Peg-IFNα group included fever (40%), myalgia (21%), fatigue (25%), alopecia (27%), neutropenia (49%), and elevated ALT (46%) (**Table 3**). One patient (1.61%) developed hyperthyroidism requiring pharmacological intervention, which resolved 24 weeks after treatment discontinuation. No severe hepatological events (including cirrhosis or HCC) were reported during the study.

**Table 3:** Adverse events associated with Peg-IFNα monotherapy or Peg-IFNα plus NAs combination therapy.

AEs	Totle (n=62)	NAs add-on Peg-IFNα group (%, n) (n=44)	Peg-IFNα group (%, n) (n=18)
Fever	64.52 (40)	68.18 (30)	55.56 (10)
Myalgia	33.87 (21)	31.82 (14)	38.89 (7)
Fatigue	40.32 (25)	40.91 (18)	38.89 (7)
Alopecia	43.55 (27)	43.18 (19)	44.44 (8)
Anxiety	14.52 (9)	13.64 (6)	16.67 (3)
Pruritus	4.84 (3)	4.55 (2)	5.56 (1)
Nausea	6.45 (4)	6.82 (3)	5.56 (1)
Neutropenia	79.03 (49)	77.27 (34)	83.33 (15)
ALT elevation	74.19 (46)	75.00 (33)	72.22 (13)
Decreased appetite	22.58 (14)	22.73 (10)	22.22 (4)
Thrombocytopenia	16.13 (10)	18.18 (8)	11.11 (2)
Thyroid dysfunction	6.45 (4)	6.82 (3)	5.56 (1)

The number and proportion of patients who experienced adverse events after receiving Peg-IFNα monotherapy or Peg-IFNα plus NAs combination therapy.

## 4 Discussion

Antiviral strategies for women with chronic HBV infection during pregnancy and postpartum require comprehensive evaluation, including treatment indications and pregnancy-related therapeutic goals. As a special population, these women also have a strong demand for functional cure of hepatitis B, which is primarily achieved with pegylated interferon alpha (Peg-IFNα)-based regimens.

Interferon has been shown to reduce HBV RNA levels, induce cccDNA degradation, and/or inhibit transcription (Zhao et al., 2024). Peg-IFNα achieves higher HBsAg clearance rates than NAs and is central to functional cure, although its efficacy varies widely across populations. Currently, functional cure is more feasible in specific “advantaged” populations (e.g., individuals with low baseline HBsAg levels, NA-experienced patients, inactive HBsAg carriers [IHCs], and pediatric patients), whereas data on early postpartum interferon therapy remain scarce (Author Group of Expert Consensus, 2025). Traditional postpartum care focuses on liver biochemistry and viral replication, with uncertain benefits of continuing, stopping, or restarting antiviral therapy (Tang et al., 2025; Liang et al., 2025). The role of immunomodulatory interferon in this period remains undefined.

This study is the first prospective multicenter investigation evaluating the efficacy and safety of Peg-IFNα therapy in this population, providing important real-world evidence for this understudied group. After 48 weeks of treatment, postpartum women with baseline HBsAg ≤ 3000 IU/mL achieved HBsAg clearance and seroconversion rates of 29.03% and 27.42%, respectively (30.43% for both after PSM), both significantly higher than the 0% in the control group. The Peg-IFNα group also showed significantly greater HBsAg reductions at weeks 24 and 48 both before and after PSM. Notably, our cohort included patients with HBsAg up to 3000 IU/mL (above the conventional 1500 IU/mL cutoff for favorable populations) as well as HBeAg- and HBV DNA-positive individuals; yet the HBsAg clearance rate was comparable to that reported in low-HBsAg, NA-experienced populations (Han et al., 2016; Ning et al., 2014; Hu et al., 2018). High rates of HBeAg seroconversion (55.56%) and complete virological response (100.00%) were also achieved. At week 24 off treatment, the functional cure rate remained stable at 32.00% (33.33% post-PSM), with only one case of HBsAg reversion during 48 weeks of follow-up. These findings indicate that the postpartum period represents a promising, clinically meaningful window for Peg-IFNα therapy and that these women constitute a functional cure-advantaged population.

The mechanism underlying the potential for achieving functional cure postpartum may be linked to immune activation (Han and Jiang, 2024). Postpartum declines in estrogen, progesterone, and human chorionic gonadotropin (hCG) levels, coupled with alterations in T lymphocyte immune profiles, may disrupt immune tolerance established during pregnancy, leading to hepatitis flares characterized by elevated ALT levels and HBV DNA fluctuations (Zhang et al., 2023). Studies confirm enhanced immune activation postpartum, with significant increased expression of multiple cytokines (Lu et al., 2023) and heightened activation of CD8^+^ T cells and subsets including effector memory T cells (TEMs) and terminally differentiated effector memory T cells re-expressing CD45RA (TEMRA) (Song et al., 2022). T-cell subsets undergo dynamic changes postpartum and exert proinflammatory effects, including an increase in Th1 subsets and a decrease in Th2 subsets, with TEMs as the predominant phenotype (Zhang et al., 2023).

Previous studies have identified both viral factors (baseline HBsAg level and its on-treatment dynamics) and host factors (age, sex, genetics, and immune status) as important predictors of HBsAg clearance (Jiang et al., 2024). Lower baseline HBsAg levels, particularly < 3.5 log10 IU/mL, are associated with higher clearance rates (Zeng et al., 2024; Zhang PX, et al., 2024). Our study supports these findings, demonstrating significantly higher HBsAg clearance in patients with baseline HBsAg < 1000 IU/mL (39.02% pre-PSM; 40.00% post-PSM). We also found that 12-week HBsAg decline was a strong predictor of HBsAg clearance at week 48 (Moucari et al., 2009; Marcellin et al., 2013), whereas baseline-to-week-24 HBsAg reduction showed no independent predictive value. This may reflect that earlier studies enrolled general populations rather than postpartum women, in whom interferon-induced immune activation may be more pronounced within the first 12 weeks. In addition, consistent with previous reports, HBsAg clearance rates did not differ significantly between Peg-IFNα monotherapy and NA combination therapy (Liu et al., 2025; Ma et al., 2020). Furthermore, 12-week ALT elevation showed no predictive value for 48-week HBsAg clearance in our study, contradicting previous reports (Wu et al., 2021).

The HBsAg clearance rate observed in our study (30.43% post-PSM) differs from that in previous reports (Lu et al., 2015; Zhong et al., 2024), likely due to several factors: (1) Later enrollment timing: Hepatitis flares postpartum occur in approximately 44.6% of women with chronic HBV infection, regardless of antiviral therapy, predominantly within the first 24 weeks postpartum (Giles et al., 2015; Kushner et al., 2018), and are especially concentrated between weeks 6 and 12 (Zeng et al., 2024; Wang et al., 2022, Wang et al., 2022). In contrast, only 2 patients in our study were enrolled within 12 weeks postpartum (both achieved HBsAg clearance). (2) Differences in baseline characteristics: The enrolled patients generally had normal ALT levels at baseline (no postpartum ALT flares), whereas baseline ALT elevation is typically associated with better treatment response (Liaw et al., 2011; Yeh et al., 2021). Our cohort also included HBeAg-positive patients and those with relatively high HBV DNA levels (up to 5 log_10_ IU/mL), whereas cohorts reporting high efficacy often included HBeAg-negative patients with HBV DNA ≤ 2000 IU/mL.

Adverse events associated with Peg-IFNα therapy require close monitoring. In addition to flu-like symptoms (fever, myalgia, and fatigue), neutropenia, thrombocytopenia, and elevated ALT levels were prominent. Furthermore, the prevalence of alopecia was relatively high, possibly related to the postpartum state and the exclusively female cohort. Although thyroid dysfunction occurred in less than 7% of patients, one patient developed drug-induced hyperthyroidism requiring medication, and thyroid function normalized 24 weeks after Peg-IFNα was discontinued.

However, this study has several limitations. First, despite its prospective multicenter design, the sample size was relatively small. The non-randomized cohort based on patient preference resulted in imbalanced enrollment and potential selection bias; large-scale randomized controlled trials are needed for validation. Second, no universal definition of the postpartum period exists. As all patients started treatment at 6–48 weeks (median 45 weeks) in this study, the narrow window prevented meaningful subgroup analysis between early and late treatment initiation. Whether different postpartum timings affect efficacy requires further investigation. Third, the lack of an external control group (e.g., non-postpartum women or those starting interferon ≥1 year postpartum) does not allow for definitive conclusions regarding whether postpartum status contributes to superior Peg-IFNα efficacy.

In conclusion, the postpartum period represents a unique therapeutic window for achieving higher functional cure rates with Peg-IFNα in women with chronic HBV infection, particularly those with low baseline HBsAg levels. This study expands existing evidence on postpartum anti-HBV management and supports integrating Peg-IFNα into personalized cure-directed strategies for this population.

## Data Availability

The raw data supporting the conclusions of this article will be made available by the authors, without undue reservation.

## References

[B1] Author Group of Expert Consensus (2025). Chinese expert consensus on clinical practice of chronic hepatitis B functional (clinical) cure. Zhonghua Gan Zang Bing Za Zhi 33, 977–987. doi: 10.3760/cma.j.cn501113-20250723-00286. PMID: . Chinese. 41167769 PMC12869179

[B2] Chinese Society of Hepatology, Chinese Medical Association Chinese Society of Infectious Diseases, Chinese Medical Association (2022). Guidelines for the prevention and treatment of chronic hepatitis B (version 2022). Zhonghua Gan Zang Bing Za Zhi 30, 1309–1331. doi: 10.3760/cma.j.cn501113-20221204-00607. PMID: . Chinese. 36891718 PMC12677433

[B3] Cui F. Woodring J. Chan P. Xu F. (2018). Considerations of antiviral treatment to interrupt mother-to-child transmission of hepatitis B virus in China. Int. J. Epidemiol. 47, 1529–1537. doi: 10.1093/ije/dyy077. PMID: 29757383

[B4] Feng Y. Yao N. Shi L. Zhu Y. Liu J. He Y. . (2023). Efficacy and safety of long-term postpartum antiviral therapy in hepatitis B virus-infected mothers receiving prophylactic tenofovir disoproxil fumarate treatment. Eur. J. Gastroenterol. Hepatol. 35, 212–218. doi: 10.1097/MEG.0000000000002476. PMID: 36574312

[B5] Ghany M. G. Pan C. Q. Lok A. S. Feld J. J. Lim J. K. Wang S. H. . (2026). AASLD/IDSA practice guideline on treatment of chronic hepatitis B. Hepatology 83, 974–997. doi: 10.1097/HEP.0000000000001549, PMID: 41186418

[B6] Giles M. Visvanathan K. Lewin S. Bowden S. Locarnini S. Spelman T. . (2015). Clinical and virological predictors of hepatic flares in pregnant women with chronic hepatitis B. Gut 64, 1810–1815. doi: 10.1136/gutjnl-2014-308211. PMID: 25431458

[B7] Han G. R. Jiang H. X. (2024). New advances in antiviral therapy during pregnancy to block mother-to-child transmission of HBV. Lin Chuang Gan Dan. Bing Za Zhi 40, 2158–2163. doi: 10.12449/JCH241105. Chinese.

[B8] Han M. Jiang J. Hou J. Tan D. Sun Y. Zhao M. . (2016). Sustained immune control in HBeAg-positive patients who switched from entecavir therapy to pegylated interferon-α2a: 1 year follow-up of the OSST study. Antivir Ther. 21, 337–344. doi: 10.3851/IMP3019. PMID: 26734984

[B9] Hu P. Shang J. Zhang W. Gong G. Li Y. Chen X. . (2018). HBsAg loss with Peg-interferon Alfa-2a in hepatitis B patients with partial response to nucleos(t)ide analog: new switch study. J. Clin. Transl. Hepatol. 6, 25–34. doi: 10.14218/JCTH.2017.00072. PMID: 29577029 PMC5862996

[B10] Huang D. X. Wang X. Y. Wang Q. Gao Y. Wang Y. Wang C. H. . (2024). Epidemiological analysis of the current prevalence of hepatitis B virus infection among pregnant and postpartum women in China from 2021 to 2023. Zhonghua Gan Zang Bing Za Zhi 32, 449–452. doi: 10.3760/cma.j.cn501113-20240422-00219. PMID: . Chinese. 38858194 PMC12898949

[B11] Hui Z. Yu W. Fuzhen W. Liping S. Guomin Z. Jianhua L. . (2024). New progress in HBV control and the cascade of health care for people living with HBV in China: evidence from the fourth national serological survey 2020. Lancet Reg. Health West. Pac. 51, 101193. doi: 10.1016/j.lanwpc.2024.101193. PMID: 39315090 PMC11419793

[B12] Infectious Diseases Physicians Branch, Chinese Medical Doctor Association Chinese Society of Infectious Diseases, Chinese Medical Association (2024). Chinese practice guidelines for the prevention and treatment of mother-to-child transmission of hepatitis B virus (version 2024). Zhonghua Gan Zang Bing Za Zhi 32, 702–711. doi: 10.3760/cma.j.cn501113-20240716-00326. PMID: . Chinese. 39267564 PMC12898883

[B13] Jiang S. Guo S. Huang Y. Yin Y. Feng J. Zhou H. . (2024). Predictors of HBsAg seroclearance in patients with chronic HBV infection treated with pegylated interferon-α: a systematic review and meta-analysis. Hepatol. Int. 18, 892–903. doi: 10.1007/s12072-024-10648-8. PMID: 38461186 PMC11126512

[B14] Kaur S. P. Talat A. Karimi-Sari H. Grees A. Chen H. W. Lau D. T. Y. . (2022). Hepatocellular carcinoma in hepatitis B virus-infected patients and the role of hepatitis B surface antigen (HBsAg). J. Clin. Med. 11, 1126. doi: 10.3390/jcm11041126. PMID: 35207397 PMC8878376

[B15] Kushner T. Shaw P. A. Kalra A. Magaldi L. Monpara P. Bedi G. . (2018). Incidence, determinants and outcomes of pregnancy-associated hepatitis B flares: a regional hospital-based cohort study. Liver Int. 38, 813–820. doi: 10.1111/liv.13594. PMID: 28941137 PMC12136009

[B16] Liang H. Liang H. Hu B. Huang L. Liang H. Su M. . (2025). Assessment of the risk of discontinuation of tenofovir disoproxil fumarate after delivery and the benefit of continued treatment in patients with immune tolerance. Front. Med. (Lausanne) 12. doi: 10.3389/fmed.2025.1597664. PMID: 41282013 PMC12634375

[B17] Liaw Y. F. Jia J. D. Chan H. L. Han K. H. Tanwandee T. Chuang W. L. . (2011). Shorter durations and lower doses of peginterferon alfa-2a are associated with inferior hepatitis B e antigen seroconversion rates in hepatitis B virus genotypes B or C. Hepatology 54, 1591–1599. doi: 10.1002/hep.24555. PMID: 22045673

[B18] Liu H. Gong H. Tan Z. Wu Y. Ran L. Mao Q. . (2025). Short-term pegylated interferon alpha in chronic HBV patients with ultra-low HBsAg: a retrospective study. Front. Cell. Infect. Microbiol. 15. doi: 10.3389/fcimb.2025.1582997. PMID: 40980017 PMC12446322

[B19] Lok A. S. F. (2024). Toward a functional cure for hepatitis B. Gut Liver 18, 593–601. doi: 10.5009/gnl240023. PMID: 38533651 PMC11249939

[B20] Lu H. Cao W. Zhang L. Yang L. Bi X. Lin Y. . (2023). Effects of hepatitis B virus infection and strategies for preventing mother-to-child transmission on maternal and fetal T-cell immunity. Front. Immunol. 14. doi: 10.3389/fimmu.2023.1122048, PMID: 36875136 PMC9978148

[B21] Lu J. Zhang S. Liu Y. Du X. Ren S. Zhang H. . (2015). Effect of Peg-interferon α-2a combined with Adefovir in HBV postpartum women with normal levels of ALT and high levels of HBV DNA. Liver Int. 35, 1692–1699. doi: 10.1111/liv.12753. PMID: 25438657

[B22] Ma Y. Wang J. Xiong F. Lu J. (2020). Extended duration therapy regimens based on Pegylated interferon for chronic hepatitis B patients focusing on hepatitis B surface antigen loss: a systematic review and meta-analysis. Infect. Genet. Evol. 85, 104492. doi: 10.1016/j.meegid.2020.104492. PMID: 32763441

[B23] Marcellin P. Bonino F. Yurdaydin C. Hadziyannis S. Moucari R. Kapprell H. P. . (2013). Hepatitis B surface antigen levels: association with 5-year response to peginterferon alfa-2a in hepatitis B e-antigen-negative patients. Hepatol. Int. 7, 88–97. doi: 10.1007/s12072-012-9343-x. PMID: 23518903 PMC3601258

[B24] Moucari R. Mackiewicz V. Lada O. Ripault M. P. Castelnau C. Martinot-Peignoux M. . (2009). Early serum HBsAg drop: a strong predictor of sustained virological response to pegylated interferon alfa-2a in HBeAg-negative patients. Hepatology 49, 1151–1157. doi: 10.1002/hep.22744. PMID: 19115222

[B25] Ning Q. Han M. Sun Y. Jiang J. Tan D. Hou J. . (2014). Switching from entecavir to PegIFN alfa-2a in patients with HBeAg-positive chronic hepatitis B: a randomised open-label trial (OSST trial). J. Hepatol. 61, 777–784. doi: 10.1016/j.jhep.2014.05.044. PMID: 24915612

[B26] Pan C. Q. Han G. R. Jiang H. X. Zhao W. Cao M. K. Wang C. M. . (2012). Telbivudine prevents vertical transmission from HBeAg-positive women with chronic hepatitis B. Clin. Gastroenterol. Hepatol. 10, 520–526. doi: 10.1016/j.cgh.2012.01.019. PMID: 22343511

[B27] Song A. Wang X. Lu J. Jin Y. Ma L. Hu Z. . (2021). Durability of hepatitis B surface antigen seroclearance and subsequent risk for hepatocellular carcinoma: a meta-analysis. J. Viral Hepat 28, 601–612. doi: 10.1111/jvh.13471. PMID: 33455067 PMC7986681

[B28] Song A. Lin X. Lu J. Ren S. Cao Z. Zheng S. . (2021). Pegylated interferon treatment for the effective clearance of hepatitis B surface antigen in inactive HBsAg carriers: a meta-analysis. Front. Immunol. 12. doi: 10.3389/fimmu.2021.779347. PMID: 34804072 PMC8600041

[B29] Song A. Liu Y. Cao Z. Lu J. Ren S. Zheng S. . (2022). Clinical features and T cell immune characteristics of postpartum hepatitis flare in pregnant women with HBeAg-positive chronic HBV infection. Front. Immunol. 13. doi: 10.3389/fimmu.2022.881321. PMID: 35493501 PMC9047935

[B30] Song M. J. (2022). Postpartum hepatic flares in immune-tolerant pregnant patients with chronic hepatitis B virus infection. Gut Liver 16, 5–7. doi: 10.5009/gnl210472. PMID: 35027508 PMC8761921

[B31] Tang Q. Wang C. Li H. Chen Z. Zhang L. Zhang J. . (2025). Unexpected HBsAg decrease after nucleoside analogues retreatment among HBeAg positive postpartum women: a pilot study. Virol. J. 22, 36. doi: 10.1186/s12985-025-02632-x. PMID: 39948654 PMC11827179

[B32] Vittal A. Sharma D. Hu A. Majeed N. A. Terry N. Auh S. . (2022). Systematic review with meta-analysis: the impact of functional cure on clinical outcomes in patients with chronic hepatitis B. Aliment. Pharmacol. Ther. 55, 1466–1472. doi: 10.1111/apt.16782. PMID: 34850415

[B33] Wang F. Song M. Hu Y. Yang L. Bi X. Lin Y. . (2022). The relation of the frequency and functional molecules expression on plasmacytoid dendritic cells to postpartum hepatitis in women with HBeAg-positive chronic hepatitis B virus infection. Front. Immunol. 13. doi: 10.3389/fimmu.2022.1062123. PMID: 36439153 PMC9681894

[B34] Wang X. Song A. Lin X. Lu J. Zheng S. Ma L. . (2022). Clinical characteristics of hepatitis flares during pregnancy and postpartum in Chinese chronic hepatitis B virus carriers-a prospective cohort study of 417 cases. Front. Immunol. 13. doi: 10.3389/fimmu.2022.1031291. PMID: 36311697 PMC9606458

[B35] Wu F. Lu R. Liu Y. Wang Y. Tian Y. Li Y. . (2021). Efficacy and safety of peginterferon alpha monotherapy in Chinese inactive chronic hepatitis B virus carriers. Liver Int. 41, 2032–2045. doi: 10.1111/liv.14897. PMID: 33896094

[B36] Yan R. Sun M. Yang H. Du S. Sun L. Mao Y. . (2025). 2024 latest report on hepatitis B virus epidemiology in China: current status, changing trajectory, and challenges. Hepatobiliary Surg. Nutr. 14, 66–77. doi: 10.21037/hbsn-2024-754. PMID: 39925891 PMC11806133

[B37] Yeh M. L. Huang J. F. Yu M. L. Chuang W. L. (2021). Hepatitis B infection: progress in identifying patients most likely to respond to peginterferon alfa. Expert Rev. Gastroenterol. Hepatol. 15, 427–435. doi: 10.1080/17474124.2021.1866985. PMID: 33338385

[B38] Zeng Z. Zhou M. F. Lin Y. J. Bi X. Y. Yang L. Deng W. . (2024). A real-world study on the features of postpartum hepatitis flares in pregnant women with chronic HBV infection. Zhonghua Gan Zang Bing Za Zhi 32, 113–118. doi: 10.3760/cma.j.cn501113-20231122-00216. PMID: . Chinese. 38514259 PMC12677309

[B39] Zhang L. Jiang T. Yang Y. Deng W. Lu H. Wang S. . (2023). Postpartum hepatitis and host immunity in pregnant women with chronic HBV infection. Front. Immunol. 13. doi: 10.3389/fimmu.2022.1112234. PMID: 36685527 PMC9846060

[B40] Zhang M. Li J. Xu Z. Fan P. Dong Y. Wang F. . (2024). Functional cure is associated with younger age in children undergoing antiviral treatment for active chronic hepatitis B. Hepatol. Int. 18, 435–448. doi: 10.1007/s12072-023-10631-9. PMID: 38376650 PMC11014810

[B41] Zhang P. X. Tang Q. Q. Zhu J. Deng W. Y. Zhang Z. H. (2024). Predictive models for functional cure in patients with CHB receiving PEG-IFN therapy based on HBsAg quantification through meta-analysis. Hepatol. Int. 18, 1110–1121. doi: 10.1007/s12072-024-10666-6. PMID: 38913149

[B42] Zhao Q. Liu H. Tang L. Wang F. Tolufashe G. Chang J. . (2024). Mechanism of interferon alpha therapy for chronic hepatitis B and potential approaches to improve its therapeutic efficacy. Antiviral Res. 221, 105782. doi: 10.1016/j.antiviral.2023.105782. PMID: 38110058

[B43] Zhong W. Yan L. Zhu Y. Shi L. He Y. Chen T. . (2024). A high functional cure rate was induced by pegylated interferon alpha-2b treatment in postpartum hepatitis B e antigen-negative women with chronic hepatitis B virus infection: an exploratory study. Front. Cell. Infect. Microbiol. 14. doi: 10.3389/fcimb.2024.1426960. PMID: 39176265 PMC11338904

